# Formulation and *in vitro*/*in vivo* evaluation of chitosan-based film forming gel containing ketoprofen

**DOI:** 10.1080/10717544.2017.1346001

**Published:** 2017-07-07

**Authors:** Dong-Won Oh, Ji-Hyun Kang, Hyo-Jung Lee, Sang-Duk Han, Min-Hyung Kang, Yie-Hyuk Kwon, Joon-Ho Jun, Dong-Wook Kim, Yun-Seok Rhee, Ju-Young Kim, Eun-Seok Park, Chung-Woong Park

**Affiliations:** a Department of Pharmacy, Chungbuk National University, Cheongju, Republic of Korea;; b Dong-a Pharmaceutical Research Laboratory, Yongin, Republic of Korea;; c Department of Pharmaceutical Engineering, Cheongju University, Cheongju, Republic of Korea;; d College of Pharmacy and Research Institute of Pharmaceutical Sciences, Gyeongsang National University, Jinju, Republic of Korea;; e College of Pharmacy, Woosuk University, Wanju-gun, Republic of Korea;; f School of Pharmacy, Sunkyunkwan University, Suwon, Republic of Korea

**Keywords:** Chitosan-based film-forming gel, chitosan, oleic acid, ketoprofen, dermal drug delivery systems, anti-inflammatory and analgesic effects

## Abstract

The film forming gel, adhered to skin surfaces upon application and formed a film, has an advantage onto skin to provide protection and continuous drug release to the application site. This study aimed to prepare a chitosan-based film forming gel containing ketoprofen (CbFG) and to evaluate the CbFG and film from CbFG (CbFG-film). CbFG were prepared with chitosan, lactic acid and various skin permeation enhancers. The physicochemical characteristics were evaluated by texture analysis, viscometry, SEM, DSC, XRD and FT-IR. To identify the mechanism of skin permeation, *in vitro* skin permeation study was conducted with a Franz diffusion cell and excised SD-rat and hairless mouse dorsal skin. *In vivo* efficacy assessment in mono-iodoacetate (MIA)-induced rheumatoid arthritis animal model was also conducted. CbFG was successfully prepared and, after applying CbFG to the excised rat dorsal skin, the CbFG-film was also formed well. The physicochemical characteristics of CbFG and CbFG-film could be explained by the grafting of oleic acid onto chitosan in the absence of catalysts. In addition, CbFG containing oleic acid had a higher skin permeation rate in comparison with any other candidate enhancers. The *in vivo* efficacy study also confirmed significant anti-inflammatory and analgesic effects. Consequently, we report the successful preparation of chitosan-based film forming gel containing ketoprofen with excellent mechanical properties, skin permeation and anti-inflammatory and analgesic effects.

## Introduction

Ketoprofen (KTP) is a nonsteroidal anti-inflammatory drug (NSAID) with analgesic and fever-reducing effects (Kantor, 1986). The analgesic effects of NSAIDs are caused by the suppression of prostaglandin synthesis by COX inhibition (Vane, 1971). Ketoprofen is a member of Class II of the Biopharmaceutical Classification System (BCS), which is characterized by high permeability and low solubility. These represent the major factor responsible for the development of a successful dermal treatment. However, oral administration is accompanied by side effects, including gastrointestinal irritation. Since ketoprofen is generally provided to the patient as long-term treatment, attempts have been made to reduce their side effects. Transdermal administration is one approach that has proved promising. In comparison to other NSAIDs, ketoprofen has a more appropriate partition coefficient and aqueous solubility for an attractive transdermal or dermal delivery candidate (Cordero et al., 1997). Dermal drug delivery system (DDS) has been exploited for the treatment of sports injuries, sprains and strains and mild acute musculoskeletal injury (Patel & Leswell, 1996). Transdermal or dermal drug delivery is also a pharmaceutical dosage form that delivers a therapeutically effective amount of the drug into the bloodstream across the skin of a patient (Rhee et al., 2008) and improve patient compliance (Cho et al., 2016). The main advantage of these systems are the release of the incorporated drug through the patient's skin into the blood circulation to achieve a controlled release manner over a long period of time (Ahmed & Khalid, 2014). Film forming gel for TDDS has studied intensively in the last decades. Lipp and Müller-Fahrnow (1999) investigated a water soluble pharmaceutical carrier gel that adhered to skin surfaces upon application and formed a film, which provided protection and drug delivery at the site of application. The researchers have demonstrated that bio-adhesive films formed from film forming polymeric materials have good properties in skin application and can provide a promising method for use in TDDS (Li et al., 2014). The dermal applications of film forming polymer is well established for providing skin protective coating and controlled drug release manner from dermal dosage form (Mundada et al., 2011). Also, the film forming gel via the dermal route could reduce unnecessary gastrointestinal absorption (El-Say et al., 2015). Recent studies have been conducted on a transdermal film of lidocaine (Padula et al., 2003), a bio-adhesive film containing oxybutynin (Nicoli et al., 2006), and a transdermal film containing tramadol (Shinde et al., 2008). Chitosan as film forming agent is a biodegradable cationic polysaccharide with anti-microbial activity (No et al., 2002) and excellent film forming ability (Domard & Domard, 2001). It can be easily grafted through reaction of the primary amino group and the primary and secondary hydroxyl groups (Vargas et al., 2009). In this study, the synthesis of chitosan-based film forming gel was conducted by direct grafting of oleic acid onto chitosan in the absence of catalysts. Grafting chitosan with oleic acid caused the improvement of film properties such as tensile strength and maximum elongation at break; these improvements are important in the application of formulations. Oleic acid is also known to be a skin penetration enhancer for polar to moderately polar molecules. The mechanisms associated with lipid phase separation have been previously proposed to account for increased skin migration (Ongpipattanakul et al., 1991). Also, the oleic acid was grafted with chitosan (Vargas et al., 2009). In this study, film forming gel containing oleic acid resulted in a 5-fold improvement of *in vitro* skin permeation rate of hairless mouse dorsal skin compared with the commercially approved product. The enhancement of skin permeability of ketoprofen by oleic acid was mainly due to disturbance of the stratum corneum (Kim et al., 1993). More specifically, oleic acid was found to increase the freedom or fluidic movement of the skin lipid structures, thereby decreasing the phase transition temperatures of the skin lipids (Walker & Smith, 1996). In addition, the prevention of drug crystallization after film formation is of importance for metastable systems such as dermal formulations. Crystallization may result in reduction in skin permeation. Therefore, the control of drug crystallization is of particular interest for the efficiency and quality of dermal systems (Ma et al., 1996). The CbFG-film containing oleic acid had an excellent inhibition effect on the crystallization of ketoprofen. The anti-inflammatory and analgesic effects of the CbFG containing ketoprofen were evaluated in a mono-iodoacetate (MIA)-induced osteoarthritis rat model (Richardson et al., 1985). In other words, *in vivo* efficacy assessment measured the degree of leg swelling degree, level of Prostaglandin E metabolite and weight distribution of each hind paw in order to evaluate the anti-inflammatory and analgesic effects. The anti-nociceptive and anti-inflammatory effect of NSAIDs in joint tissue is attributed to Prostaglandin E2 reduction in the inflamed tissue, which was determined by the level of Prostaglandin E metabolite. Prostaglandin E2 production in the synovial fluid is rapidly metabolized to more a stable form, which allowed the estimation of the amount of drug release (Cialdai et al., 2009).

## Materials and methods

### Materials

Ketoprofen (USP Reference Standard) was purchased from Sigma Aldrich^®^. Chitosan (Koyo Chemical, Sakai-minato, Japan; degree of deacetylation, 95%; M.W., 200,000 Da) and lactic acid (Chitosan; Koyo Chemical, Sakai-minato, Japan) were a gift from Dong-a Pharmaceutical Co. Ltd (Kyungki-do, Korea). Propylene glycol (biotech grade, >99.5%) was purchased from Georgia Tech (Atlanta, GA), and oleic acid was purchased from Samchun chemical Co. Ltd (Pyeongtaek, Korea). All the other chemicals were reagent grade or higher. Water was purified by reverse osmosis and filtered in-house. The commercial products were Ketotop^®^ gel (HANDOK LTD., Korea), kenofen^®^ gel (ILDONG Pharm. Co., LTD., Korea).

### Preparation of chitosan-based film forming gel containing ketoprofen

To prepare the chitosan-based film forming gel containing ketoprofen, chitosan was first dispersed in water during 1 min using mechanical homogenizer (T-18 Ultra-Turrax, IKA Ltd, Staufen, Germany). This dispersed chitosan suspension was pre-mixed with ketoprofen, oleic acid, propylene glycol, ethyl alcohol, and various skin permeation enhancers such as oleic acid (OA), tween^®^ 80 (Tw80), *n*-methylpyrrolidone (NMP) and Cremophor^®^ RH 40 (Cr40). Then, the pre-mixture was homogenized for 15 min using mechanical homogenizer (T-18 Ultra-Turrax, IKA Ltd, Staufen, Germany). The required viscosity, prepared by the addition of lactic acid, was confirmed by viscometer (DV-2 T, Brookfield Ltd, Middleborough, MA). After the confirmation, the CbFG was prepared. The composition in this study is shown in Table 1.

**Table 1.** Formulation of CbFG containing permeation enhancer.

weight ratio %	CbFG-OA 5%	CbFG-NMP 5%	CbFG-Tw80 5%	CbFG-Cr40 5%	CbFG-OA 3%	CbFG-OA 2%	CbFG-OA 1%	CbFG-OA 0.5%
Ketoprofen	3							
Chitosan	3							
Lactic acid	4							
Propylene glycol	2							
Oleic acid	5	–	–	–	3	2	1	0.5
NMP	–	5	–	–	–	–	–	–
Tween 80	–	–	5	–	–	–	–	–
Cremophor RH 40	–	–	–	5	–	–	–	–
95% Ethanol	40	40	40	40	40	40	40	40
D.W.	43	43	43	43	45	46	47	47.5
Total	100							

### Physicochemical evaluation of CbFG and CbFG-film

#### Scanning electron microscopy (SEM)

Morphology of CbFG and CbFG-film was observed by SEM. CbFG were casted onto cover glass and dried in oven at 40 °C for 30 min until the film was formed completely. The films were coated with a gold film (600 Å) using a sputter deposition technique and observed in a scanning electron microscope (JSM-7600 F, JEOL, Tokyo, Japan).

#### Texture analyzer

The maximum elongation at break and tensile strength were evaluated by the Texture Analyzer (TA.XT *plus*, Stable Micro Systems Ltd., Surrey, UK) (Hurler et al., 2012) and calculated with software (Exponent, Stable Micro Systems, Ltd., Surrey, UK). CbFG was casted on a polyester film, and oven-dried at 40 °C for 30 min until the CbFG-film was formed completely. The dimensions (width × length) of each film were 20 mm × 60 mm and the thickness was 50 μm. The distance between the supports was set at 40 mm, and the crosshead speed was fixed at 1 mm/min. The tensile strength and maximum elongation at break were calculated for the film, which was cut in two directions longitudinally.

#### X-ray powder diffraction (XRD)

The diffraction pattern of the CbFG-film was analyzed by D8 Discover with GADDS (Bruker AXS Inc., Fitchburg, WI; XRD). The 2θ scans were performed between 5° and 60° with a scan interval of 0.1°. The crystallinity of ketoprofen was analyzed by changes in intensity of the diffraction pattern of CbFG-film.

#### Fourier transform infrared spectroscopy (FT-IR)

ATR FT-IR spectroscopy was performed on chitosan and CbFG-films in the range 650–4000 cm^−1^, using an IFS 66/S spectometer (BRUKER OPTIK GMBH, Germany). The CbFG-film containing ketoprofen was measured. CbFG was casted on a polyester film, and oven-dried at 40 °C for 30 min. A part of polyester film taken and evaluated.

#### Differential scanning calorimetry (DSC)

The thermal response of the prepared CbFG-film was analyzed using a DSC Q2000 (TA Instruments, Saugus, MA) thermal analyzer system. Prepared CbFG-films were weighed, loaded in an aluminum pan and analyzed with a heating rate of 10 °C/min over a temperature range of 30–200 °C. Approximately 3 mg of sample was loaded into a standard DSC pan, and the thermal response was calculated with Universal Analysis Software v5.2.6 (TA Instruments).

### *In vitro* skin permeation study

The *in vitro* skin permeation profile of CbFG-film was evaluated in SD-rat and hairless mouse dorsal skin, using a Franz diffusion cell. The dorsal skin of SD-rat and nude mice were harvested and the subcutaneous fat was removed. The skin was mounted directly in the Franz diffusion cell (FCDS-900 °C, Fine science tools Ltd, Foster City, CA) and each topical product was applied to a part of the skin (1.1 cm^2^ for gel preparations). The receptor phase (PBS, pH 7.4, 12 mL) was collected for 12 h, and the study was conducted at 37 °C. PBS is buffer solution commonly used in biological research. The osmolarity and ion concentrations of the solutions match those of the human body. After centrifugal filtration, the supernatant was used to determine the concentration of the test drug by liquid chromatography (Thermo Fisher Scientific Co. Ltd, Waltham, MA).

### *In vivo* efficacy assessment

#### Animals

Eight-week-old male Sprague–Dawley (SD) rats were obtained from Orient Bio Ltd. (Kyungki-do, Korea). The SD rats were cultivated in an environment with a controlled light cycle (12 h light:dark) and controlled temperature (23 ± 1 °C). Tap water and standard laboratory feed were available *ad libitum*. SD rats were domesticated at least three days before treatment. All animal experiments were performed in accordance with the ‘Principles of Laboratory Animal Care’ and were approved by the Committee for Animal Experiments of Chungbuk National University (Cheongju, Korea).

#### Monoiodoacetate-induced osteoarthritis model

The monoiodoacetate (MIA)-induced arthritis model in rats was used (Fernihough et al., 2004). Male SD rats were anesthetized with isoflurane, and then injected with MIA (3 mg in 30 μL saline) into the right knee. A saline injection was used in the positive control group.

#### Joint swelling measurement

Two days after MIA treatment, each formulation was administered three times a day for 4 days. The resulting anti-inflammatory effect, as demonstrated by a decrease in knee swelling, was measured with digimatic electronic calipers (Mitutoyo, Japan) at 2 and 6 days after treatment, and represented by the asymmetry of knee diameters between the ipsilateral and contralateral knee joints (Ashraf et al., 2011).

(1) % Joint swelling inhibition=[1-(Positive group joint diameter÷ Application group joint diameter)] ×100

### Pain assessment

CbFG-film-OA was characterized by changes in weight distribution on each hind paw (Yoshimi et al., 2010). Thus, a hind limb weight-bearing apparatus (incapacitance tester; Linton Instrumentation, Norfolk, UK) was used to assess the difference in the distribution of weight between the right (osteoarthritic) and the left (contralateral control) hind limbs at 72 h (Yoshimi et al., 2010) Animals were placed in a balance chamber on a plate to weigh each hind limb, and were allowed to acclimatize to the apparatus. When stationary, the force exerted on the plate by each hind paw was recorded over a period of 3 s and force unit was grams. The measurement was repeated four times, and the readings for each rat at each time point were used to calculate the average value. The study was carried out in blinded manner, and the percentage weight distribution of the right leg was calculated by the following equation.

(2) % Weight of right leg =100×[Right limb weight÷ (Right limb weight + Left limb weight)]

### Prostaglandin E2 metabolite measurement

The level of prostaglandin E2 metabolite in the joint was measured by a modification of a previously described method (Magari et al., 2003). In brief, the right knee of each rat was cleaved using a bone cutter and stored at −70 °C until use. The tissue was soaked in 5 mL of saline, homogenized on ice using a high-speed homogenizer (T-18 Ultra-Turrax, Ika, Germany), and incubated on ice for a further 3 days. The material was centrifuged at 13000 rpm for 10 min and the supernatant was collected. The concentration of Prostaglandin E2 metabolite was determined by ELISA following the manufacturer’s instructions (Cayman Chemical Company, 514531).

### High performance liquid chromatography (HPLC) analysis

The prepared CbFG films were analyzed with an HPLC system (Thermo Fisher Scientific Co. Ltd, Waltham, MA) consisting of an isocratic pump, an auto sampler, a column oven, a UV detector (Ultimate 3000) and HPLC System software; the detection wavelength was 233 nm. The analytical column was 4.6 mm ×25 cm, packing L1. The mobile phase consisted of a solution of ACN:DW:Buffer in a volume ratio of 490:430:80 delivered at a rate of 1 mL/min. The injection volume of each sample was 20 μL and the column temperature was maintained at 25 °C. This method is based on the ketoprofen assay method of USP. The inter- and intra-data of the HPLC are shown in Table 2.

**Table 2.** Inter- and Intra-data on ketoprofen analysis using HPLC.

Concentration of Ketoprofen (μg/ml) (*n* = 3)	Inter day		Intra day	
	Mean ± SD	% RSD	Mean ± SD	% RSD
15.60	15.72 ± 0.20	1.25	15.34 ± 0.08	0.53
62.50	62.26 ± 0.35	0.57	62.76 ± 0.04	0.07
250.00	249.71 ± 0.94	0.38	249.62 ± 0.03	0.01

### Statistical analysis

One-way analysis of variance (ANOVA) tests were employed using Statistics Software (SPSS, IBM Ltd, Armonk, NY) to determine statistical significance. The results were presented as the mean ± SD. Differences were considered statistically significant for values of *p < 0.05 and **p < 0.01.

## Results and discussion

### Preparation of CbFG and CbFG-films containing ketoprofen

**Figure 1.**
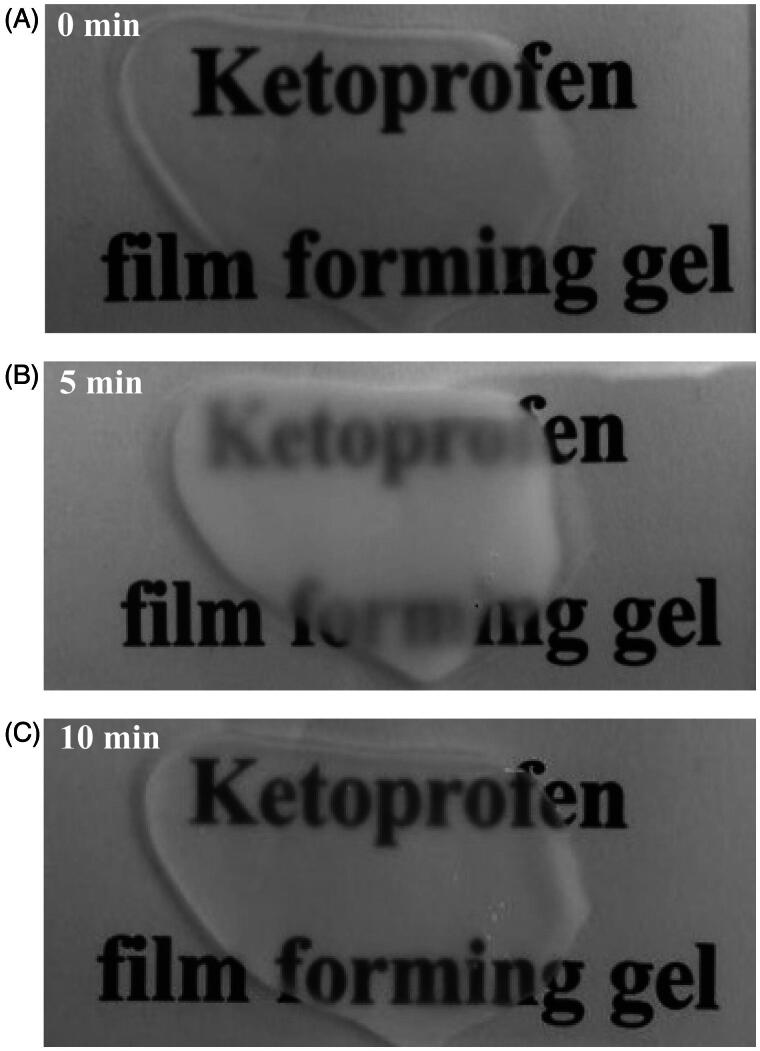
Transparency changes of CbFG-film during film formation; (A) initial state after CbFG application, (B) 5 min after CbFG application; (C) 10 min after CbFG application.

Table 1 shows that compositions of CbFG, which basically contained 3% w/w chitosan and 2% w/w propylene glycol for the appropriate viscosity formation. At first, the viscous and transparent chitosan dispersion was prepared by the addition of lactic acid. After that, CbFG were prepared with other excipients and the prepared CbFG were opaque without any foreign substance. The viscosity and gel properties of CbFG films with or without oleic acid were evaluated. The viscosity was approximately 5000 cP. There was no significant difference in the viscosity and gel properties between CbFG films and commercial products. As shown in Figure 1, the change of transparency in the course of CbFG becoming a CbFG-film after gel application. The CbFG was transparent initially after application, but it quickly turned opaque state and, when the gel was completely dried after 10 min, the CbFG-film changed transparent.

### Mechanical properties of CbFG-films

**Figure 2.**
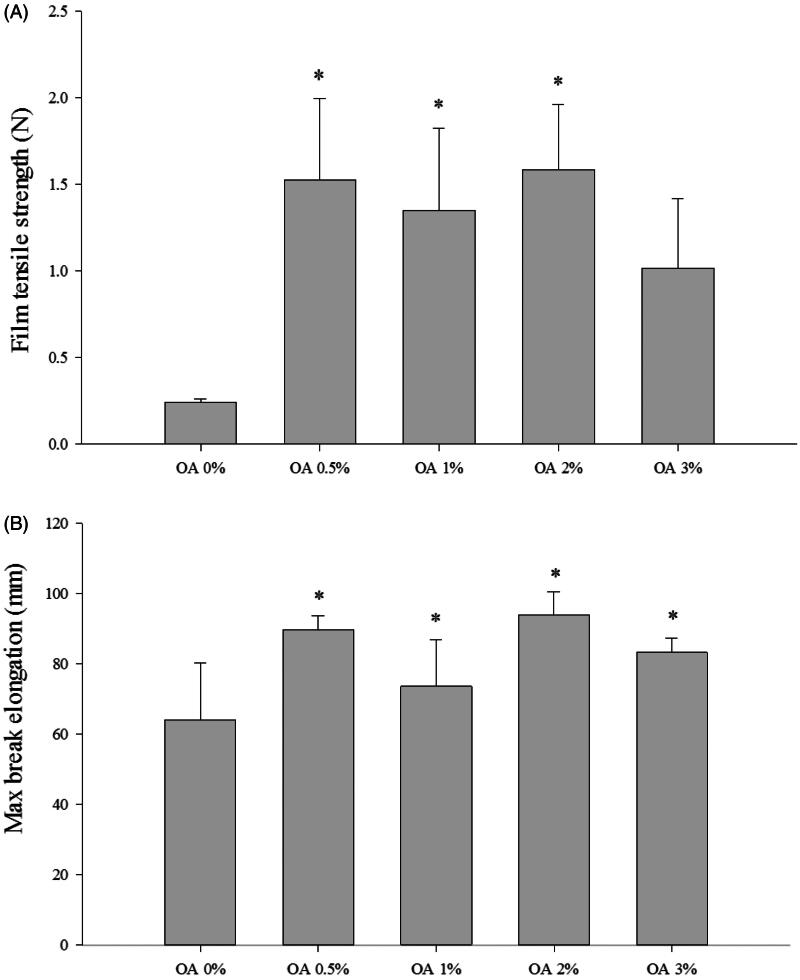
Mechanical properties of CbFG-films; (A) film tensile strength, (B) maximum elongation at break. One-way ANOVA analysis, *p < 0.05 (*n* = 4) compared with film containing OA 0%.

Figure 2(A,B) shows the tensile strength of the films. It was observed that the inclusion of oleic acid led to an increase in the modulus of elasticity and tensile strength, as well as an increase in the maximum elongation at break. The changes were statistically significant when considering both the tensile strength and maximum elongation at break. The formulation based on the mixture of chitosan and oleic acid resulted in an improvement in film properties when compared to CbFG-film without oleic acid. In addition, the result of the maximum elongation at break is a measurement of the flexibility of the film and it is defined as the ability of the film to deform before breaking. The film containing oleic acid was much less brittle than the CbFG-film without oleic acid, because the maximum elongation at break of the film with oleic acid was higher than CbFG-film without oleic acid. This could be attributed that the addition of oleic acid was sufficient to promote mechanical improvements in the CbFG-films. This phenomenon could be due to the interaction between chitosan and oleic acid, which reduced, or even prevented, the deformation of film by covalent bonds between them (Nascimento et al., 2012).

### Physicochemical evaluation of CbFG-films

#### Surface morphologies of CbFG-films

**Figure 3.**
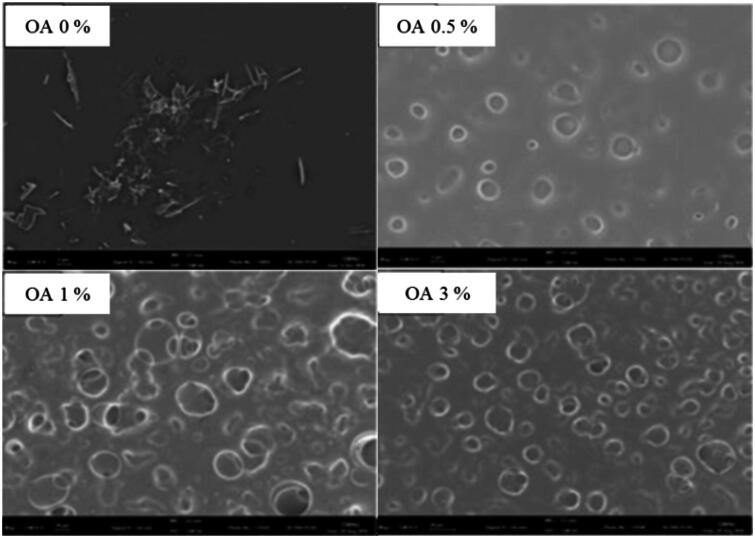
SEM micrographs of CbFG-films without or with OA.

The surface morphologies of CbFG-film without oleic acid were compared with the films containing oleic acid using SEM (Figure 3), and the surface structures showed remarkable differences. A needle-shape crystalline of ketoprofen was observed for the CbFG-film excluding oleic acid; in contrast, while a smooth, continuous surface was observed for the Chitosan film surface, the presence of oleic acid caused discontinuities associated with the formation of the two phases of globules and background in the film. The increase of the content of oleic acid in the film led to an appreciable difference, demonstrating the increase of globule numbers and size on the film surface. The creation of these globules was attributed to the grafting of the chitosan and oleic acid (Vargas et al., 2009). The reaction of chitosan with oleic acid was catalyzed which reacts with carboxyl groups of oleic acid to from an active ester intermediates. Consequently, the intermediates can react with primary amino groups of chitosan to form an amide bond (El Fray et al., 2012). Figure 3 also shows the possibility of crystal inhibition of the CbFG film containing oleic acid. The solubility of ketoprofen in water, ethanol and oleic acid was reported 51 μg/mL, 100 mg/mL and 24 mg/mL (Kim & Choi, 2002). In the film state, water and ethanol was dried, leaving only oleic acid, ketoprofen was crystallized in the film without oleic acid, but when oleic acid was added, crystallization of ketoprofen was prevented by solubility capacity of oleic acid, and ketopropen was expressed to position in the globular particles formed by the oleic acid (Yahya & Arof, 2003).

#### FTIR spectra of CbFG-films

**Figure 4.**
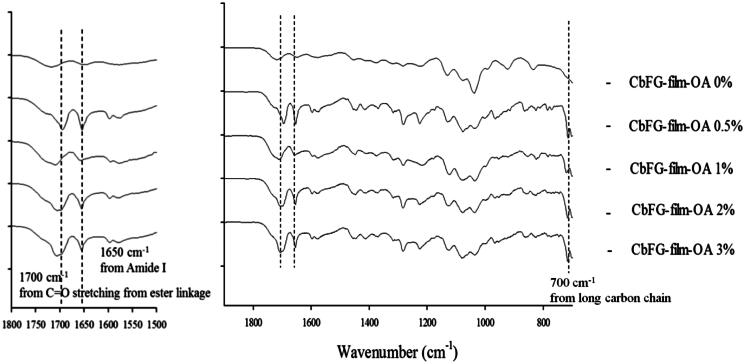
FT-IR spectra of CbFG-films without or with OA.

The FTIR spectra of the samples are shown in Figure 4. For the spectrum of the CbFG-film without oleic acid, the characteristic absorption bands appeared at 1650 cm^−1^ (amide I), 1578 cm^−1^ (amide II) and 1720 cm^−1^ (C=O bond of the carboxyl groups of lactic acid). In contrast, in case of CbFG-films with oleic acid 0.5, 1, 2 and 3%, the characteristic adsorption band of the long-chains in oleic acid appeared at 700 cm^−1^ only, intensity of the peak at 1650 cm^−1^ of amide I was increased, and other sharp bands were observed at 1280, 1592 and 1700 cm^−1^. However, Films containing oleic acid does not show any absorption peaks of C=O stretching from the ester linkage, which are normally found in the range 1720–1740 cm^−1^. Instead, the original amide peak (amide I) increased in intensity after reaction and shifted to lower wavenumber 1700 cm^−1^ due to hydrogen bonding. The 1592 cm^−1^ peak of –NH_2_ bending vibration of chitosan also blue-shifted to 1570 cm^−1^ (Li et al., 2008).

#### XRD diffractograms of CbFG-films

**Figure 5.**
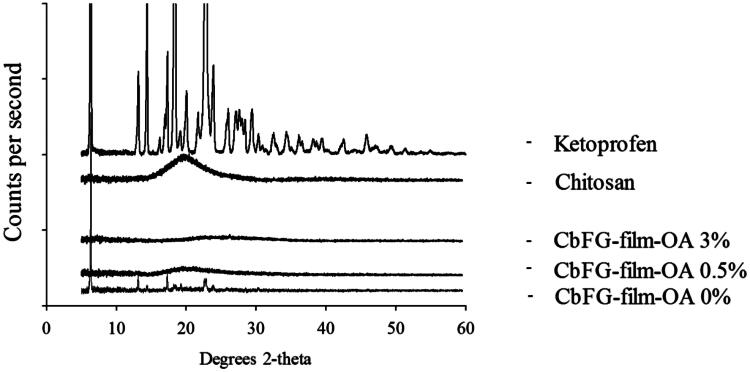
XRD diffractograms of CbFG-films without or with OA, raw chitosan and raw ketoprofen.

The crystallinity of CbFG-films were identified using XRD in Figure 5. The diffraction peaks of CbFG-film with or without oleic acid were compared with the ketoprofen crystal peak, which has a high crystallinity, which represented as a high intensive diffraction peak in specific 2-theta value such as 6.2, 13.2, 17.5° and many other things. The diffraction peak of the CbFG-films with oleic acid 0.5% and 3% demonstrated the films maintained an amorphous form, which could be induced by oleic acid. In contrast, the CbFG-films without oleic acid had a diffraction peaks at 6.2, 13.2 and 17.5°. Oleic acid had a potential to inhibit a crystallization of ketoprofen during CbFG-film formation, and as described above, it could be explained with the grafting of chitosan and oleic acid. The morphological observation of formation of ketoprofen crystal could be mainly due to the preferential adsorption of deprotonated oleic acid, which changes the surface energy of crystal planes and leads to different growth rates along corresponding directions (Bu et al., 2009). Furthermore, each XRD diffractogram of CbFG-films with oleic acid displayed a halo peak at a 2-theta value of around 20°, which represented the arrangement of chitosan molecules in CbFG-films. This phenomenon is more pronounced when sample water activity increases, which indicates a promotion of lipid binding with an increase in the molecular mobility (Fabra et al., 2010).

#### DSC thermograms of CbFG-films

**Figure 6.**
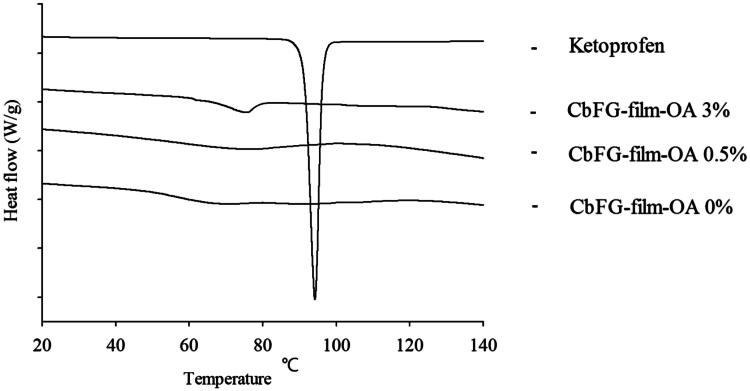
DSC thermograms of CbFG-films without or with OA and raw ketoprofen.

In case of CbFG films with oleic acid 0, 0.5 and 3%, the endothermic peak of chitosan was observed in the range of 60–80° on all CbFG-films at (Figure 6). According to the reference, it was reported as an endothermic peak derived from chitosan (Koosha et al., 2015). Chitosan peaks at around 60–80 had a tendency to shift slightly as the proportion of oleic acid increases, which is seen as an effect between the chitosan and oleic acid (Ruhi et al., 2015). The endothermic melting peak of ketoprofen was observed at 94 °C, which was not shown with CbFG films with oleic acid 0, 0.5 and 3% because the films had a mixture of several substances.

### *In vitro* skin permeation study

**Figure 7.**
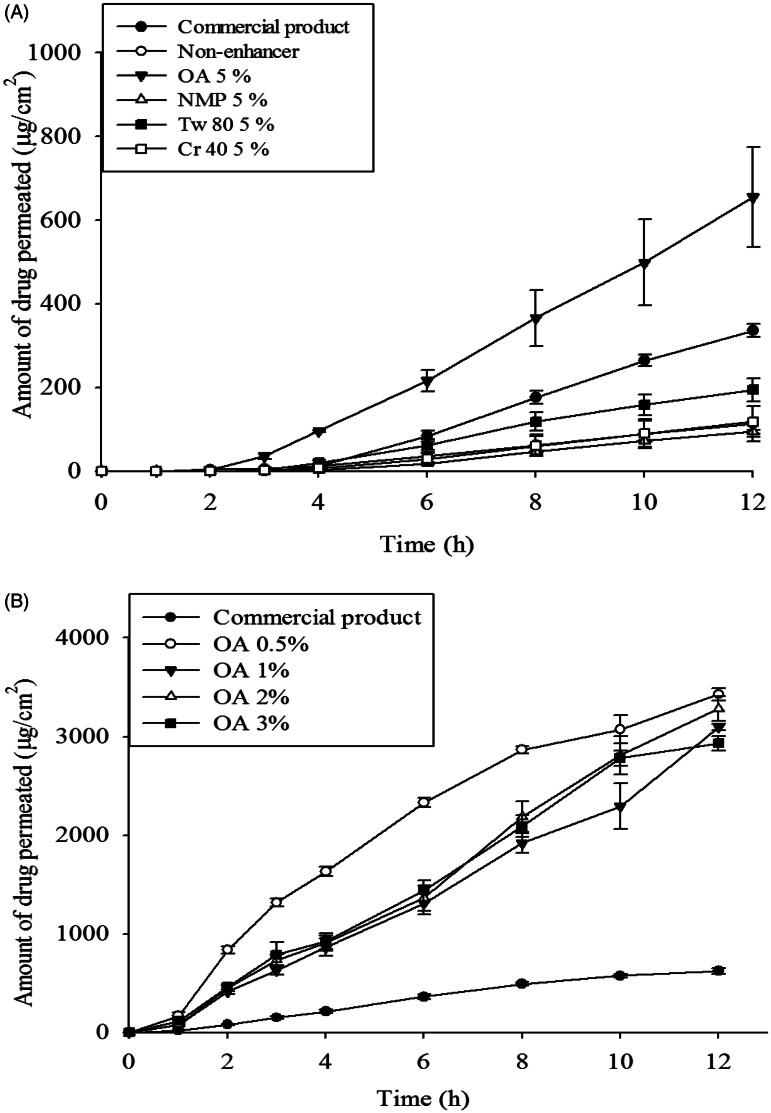
*In vitro* skin permeation profiles of CbFG-film containing ketoprofen; (A) with different kinds of enhancers through excised SD-rat skin, (B) with different amount oleic acid as an enhancer through excised hairless mouse skin. One-way ANOVA analysis,* *p* < 0.05 (*n* = 3) compared with Commercial product.

As shown in Figure 7, CbFG-films increased the ketoprofen permeation rate during the 12 h *in vitro* skin permeation study. The presence or absence of oleic acid in the CbFG film significantly affected dermal delivery of ketoprofen. The permeated amount of ketoprofen was significantly increased in CbFG films with 5% of oleic acid compared to other permeation enhancer candidates (Figure 7A), which indicated the composition of oleic acid influenced the skin permeation activity of CbFG-films. The flux and lag time of CbFG containing various permeation enhancers was described (Table 3). Flux of CbFG containing OA was 75.4 ± 5.0 μ/cm^2^/h, the flux of CbFG containing Tw80, NMP and Cr40 was each 24.3 ± 1.5, 13.6 ± 1.0 and 15.2 ± 3.3 μg/cm^2^/h in SD rat skin. The use of CbFG –OA 0.5% resulted (flux 308.6 ± 11.5 μg/cm^2^/h) in a 3-fold improvement in compared with the commercial drug (flux 95.5 ± 28.0 μg/cm^2^/h) in hairless mouse skin. This can be explained by the disturbance of the stratum corneum by oleic acid (Kim et al., 1993). Also, the penetration enhancers containing unsaturated alkyl chains, then C18 appears near optimum. For such unsaturated compounds, the bent *cis* configuration is expected to disturb intercellular lipid packing more so than the *trans* arrangement, which differs little from the saturated analog (Williams & Barry, 2012). Permeation enhancers such as OA which reduce the diffusional resistance of the skin by interacting with the lipid matrix, have been postulated to act by increasing lipid fluidity (Naik et al., 1995). Another possible mechanism for the action of OA is lamellar solid fluid phase separation. When applied together with ethanol, OA is also believed to cause stratum corneum lipid extraction (Touitou et al., 2002). Alteration of the percentage of oleic acid resulted in no significant difference in the accumulated drug amount (Figure 7(B) and Table 3). However, the skin permeation rate of the CbFG film containing 0.5% oleic acid is faster than high oleic acid contents due to the small surface particles (Figure 7(B)).

**Table 3.** *In vitro* skin permeation rate and lag time of CbFG-film through excised SD-rat skin and hairless mouse skin.

	Commercial product		CbFG Non enhancer	CbFG OA 5% *		CbFG NMP 5%		CbFG Tw80 5%		CbFG Cr40 5%
*In vitro* skin permeation rate through excised SD-rat skin										
Flux (μg/cm^2^/h)	39.9 ± 6.1		12.6 ± 5.1	75.4 ± 5.0		13.6 ± 1.0		24.3 ± 1.5		15.2 ± 3.3
Lag time (h)	3.3 ± 1.7		3.2 ± 1.3	3.7 ± 0.8		2.8 ± 0.3		3.4 ± 0.8		4.1 ± 0.7
	Commercial product	CbFG OA 3%*			CbFG OA 2%*		CbFG OA 1%*		CbFG OA 0.5%*	
*In vitro* skin permeation rate through excised hairless mouse skin										
Flux (μg/cm^2^/h)	95.5 ± 28.0	209.9 ± 17.6			281.7 ± 77.0		264.9 ± 7.3		308.6 ± 11.5	
Lag time (h)	1.7 ± 0.1	1.7 ± 0.9			1.8 ± 0.4		1.5 ± 0.5		1.0 ± 0.6	

One-way ANOVA analysis.

* *p* < 0.05 (*n* = 3) compared with Commercial product.

### Therapeutic efficacy of the CbFG-films in SD rats with experimentally induced OA

**Figure 8.**
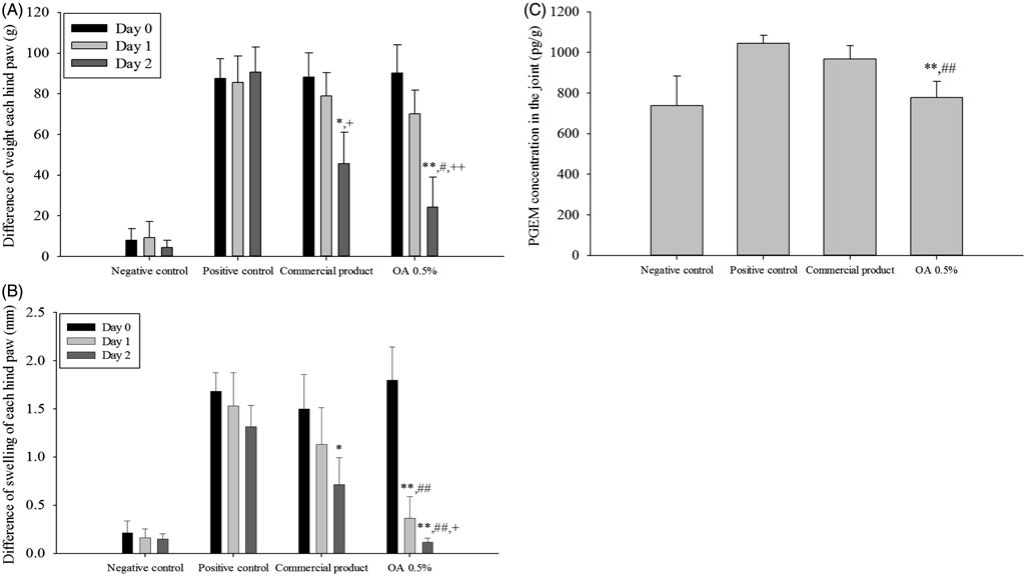
*In vivo* efficacy study of CbFG-film-OA 0.5% and commercial product; (A) difference in swelling of each hind paw, (B) difference in weight of each hind paw, (C) PGEM levels in joint tissue for 2 days after administration. One-way ANOVA analysis: *p < 0.05 (*n* = 6), compared with positive control; **p < 0.05 (*n* = 6), compared with positive control; #*p* < 0.05 (*n* = 6), compared with negative control; ##*p* < 0.05 (*n* = 6), compared with negative control; +*p* < 0.05 (*n* = 6), compared with Commercial product; ++*p* < 0.01 (*n* = 6), compared with Commercial product.

The X-ray data (data not shown) showed progress without significant damage of MIA-induced arthritis model in rats for experimental period. During the test period, animals weighted about 250 g, indicating that proper drug application was achieved without gastrointestinal disorder. The anti-inflammatory effect of the CbFG film on joint swelling is shown in Figure 8(A). Significant joint swelling was observed 1 day after application of MIA (3 mg/30 μL) in the right knee, with an increase of approximately 2 mm in the right knee joint diameter compared with the negative control group. The diameter of the knee joint was only decreased about 8% over a further 72 h in the positive control group. In contrast, application of both the commercial product and CbFG film containing 0.5% oleic acid resulted in marked improvements, resulting in approximately 26 and 76% on the first day of application decrease in knee joint swelling inhibition %, respectively, compared with the positive control group in (Figure 8(A)). There was a significant difference between commercial product application group and CbFG application group. We assessed the weight asymmetry of the injured hind paw and noninjured hind paw as index to assess the analgesic effect of CbFG-films. The weight difference of each hind paw during the application period was shown in Figure 8(B). Weight asymmetry in the experimental rat induced of arthritis was thought to be due to the pain accompanied by the damage of cartilage. The rats in the MIA injected groups showed weight asymmetry with values of 27–28%, whereas rats in the negative control group showed weight symmetry. A comparison of the application of the commercial product and CbFG film containing 0.5% oleic acid showed greater values at 2 day after drug application (about each 38%, 44%), suggesting an analgesic effect. However, there was a significant difference between commercial product application group and CbFG application group. The level of Prostaglandin E2 metabolite in the joint tissue was determined as the anti-inflammatory effect of NSAIDs is understood to be caused by the decrease in Prostaglandin E2 in the inflamed tissue. Because Prostaglandin E2 produced in the synovial fluid is rapidly converted *in vivo* to its more stable metabolites, an estimate of the amount released was quantified using the previous reported method (Park et al., 2014). Topical application of the commercial product and CbFG film with 0.5% oleic acid increased Prostaglandin E2 levels in the joint by 7 and 18%, respectively, compared with the positive control group (Figure 8(C)). This could be due to the dermal-delivered therapeutic effect of ketoprofen.

## Conclusion

In this study, we aimed to prepare a chitosan-based film-forming gel that is continuously present on the surface of the skin after application and intended for sustained release, and used chitosan to control the film formation as a functional excipient. The grafting of chitosan onto oleic acid performed a key role in the inhibition of crystal formation of ketoprofen in the CbFG-films as well as physicochemical and biological investigations performed in this study suggest that oleic acid caused modulation on polymeric globules on the CbFG-films, leading to remarkable increase of *in vitro* skin permeation ability of CbFG-films and improved swelling inhibition, weight asymmetry and prostaglandin E2 inhibition of ketoprofen in MIA-induced model. Additionally, film properties of CbFG-films were excellent. These results strongly suggest that chitosan-based film forming gel holds a promising potential in various aspects as a topical DDS system in the pharmaceutical industries to increase the utility of various bioactives. In conclusion, CbFG has excellent physicochemical properties compared with the commercial product. The important component is oleic acid and this phenomenon was investigated by SEM, FT-IR, DSC, XRD and *in vitro* and *in vivo* efficacy studies.
